# Complete Genome Sequences of Two Novel KPC-2-Producing IncU Multidrug-Resistant Plasmids From International High-Risk Clones of *Escherichia coli* in China

**DOI:** 10.3389/fmicb.2021.698478

**Published:** 2021-07-21

**Authors:** Wenhao Wu, Lingling Lu, Wenjia Fan, Chun Chen, Dazhi Jin, Hongying Pan, Xi Li

**Affiliations:** ^1^Department of Infectious Diseases, Zhejiang Provincial People’s Hospital, People’s Hospital of Hangzhou Medical College, Hangzhou, China; ^2^Medical College, Qingdao University, Qingdao, China; ^3^Adicon Clinical Laboratories, Hangzhou, China; ^4^Department of Pneumology, Zhejiang Provincial People’s Hospital, People’s Hospital of Hangzhou Medical College, Hangzhou, China; ^5^Centre of Laboratory Medicine, Zhejiang Provincial People’s Hospital, People’s Hospital of Hangzhou Medical College, Hangzhou, China

**Keywords:** *E. coli*, KPC-2, IncU plasmid, high-risk clones, whole genome sequencing

## Abstract

The rapidly increasing prevalence of *Klebsiella pneumoniae* carbapenemase 2 (KPC-2)-producing bacteria has become a serious challenge to public health. Currently, the *bla*_*KPC–*__2_ gene is mainly disseminated through plasmids of different sizes and replicon types. However, the plasmids carrying the *bla*_*KPC–*__2_ gene have not been fully characterized. In this study, we report the complete genome sequences of two novel *bla*_*KPC–*__2_-harboring incompatibility group U (IncU) plasmids, pEC2341-KPC and pEC2547-KPC, from international high-risk clones of *Escherichia coli* isolated from Zhejiang, China. Two KPC-2-producing *E. coli* isolates (EC2341 and EC2547) were collected from clinical samples. Whole-genome sequencing (WGS) analysis indicated that EC2341 and EC2547 belonged to the ST410 and ST131 clones, respectively. S1-nuclease pulsed-field gel electrophoresis (S1-PFGE), Southern blot and conjugation experiments confirmed the presence of the *bla*_*KPC–*__2_ gene on the pEC2341-KPC plasmid and that this was a conjugative plasmid, while the *bla*_*KPC–*__2_ gene on the pEC2547-KPC plasmid was a non-conjugative plasmid. In addition, plasmid analysis further revealed that the two *bla*_*KPC–*__2_-harboring plasmids have a close evolutionary relationship. To the best of our knowledge, this is the first report of *E. coli* strains carrying the *bla*_*KPC–*__2_ gene on IncU plasmids. The emergence of the IncU-type *bla*_*KPC–*__2_-positive plasmid highlights further dissemination of *bla*_*KPC–*__2_ in *Enterobacteriaceae*. Therefore, effective measures should be taken immediately to prevent the spread of these *bla*_*KPC–*__2__–_positive plasmids.

## Introduction

The rapidly increasing prevalence of KPC-producing bacteria has become a serious challenge to public health (Suay-García and Pérez-Gracia, 2019). At the time of writing (April 2021), 82 variants of KPC enzymes (KPC-1 to KPC-82) have been identified among gram-negative bacteria worldwide^1^. Among these carbapenemases, KPC-2 was first identified from a *Klebsiella pneumoniae* strain in the United States in 2003 (Smith Moland et al., 2003) and attracted extensive attention because of its rapid worldwide dissemination. Currently, the *bla*_*KPC–*__2_ gene is prevalent in *K. pneumoniae* strains, and the sequence type 258 (ST258) clone has successfully spread worldwide (Munoz-Price et al., 2013).

Although not as common as in *K. pneumoniae*, the *bla*_*KPC–*__2_ gene has also been identified in *Escherichia coli* strains. Some reports, including two from our group, have recently found that the *bla*_*KPC–*__2_ gene was present in the ST131-type *E. coli* strains, which are international multidrug-resistant high-risk clones (Du et al., 2020; Wang et al., 2020). KPC-2-producing *E. coli* strains were isolated not only from humans but also from animals, such as cattle (Vikram and Schmidt, 2018), swine (Liu et al., 2018) and cats (Sellera et al., 2018). Unfortunately, *bla*_*KPC–*__2_ has also been identified in environmental samples [urban rivers (Xu et al., 2015), drinking water (Mahmoud et al., 2020), and vegetables (Wang et al., 2018)], indicating its presence in the environment. In addition, *bla*_*KPC–*__2_ was further disseminated through plasmids of different sizes and replicon types (Mathers et al., 2017), such as the pKpQIL-like plasmid (Chen et al., 2014b), the IncFIA plasmid (Chen et al., 2014a), the IncI2 plasmid (Chen et al., 2013), the IncX3 plasmid (Fuga et al., 2020), the IncP-6 plasmid (Hu et al., 2019) and the IncN plasmid (Schweizer et al., 2019). The movement of *bla*_*KPC*_ plasmids into *E. coli* strains that are known pathogens of urinary tract and intra-abdominal infections raises clinical concerns (Bratu et al., 2007). Plasmid transfer will further lead to continued spread of resistance and limit clinical treatment options (Chen et al., 2014). However, plasmids carrying the *bla*_*KPC–*__2_ gene have not been fully characterized.

In the present study, we reported the complete sequences of two novel *bla*_*KPC–*__2_-harboring IncU plasmids from international high-risk clones of *E. coli* ST131 and ST410 isolates from China. In addition, the whole genome sequence revealed that the two *bla*_*KPC–*__2_-positive plasmids have a close evolutionary relationship.

## Materials and Methods

### Bacterial Strains

In a retrospective study, 109 carbapenem-resistant *Enterobacteriaceae* strains were isolated from June 2018 to September 2019. Common carbapenemase genes (*bla*_*KPC*_, *bla*_*NDM*_, *bla*_*VIM*_, and *bla*_*IMP*_) were amplified, and the positive products were sequenced. Two KPC-2-producing *E. coli* strains were included in this study and further identified by the VITEK MS system (bioMérieux, Marcy-l’Etoile, France).

### Antimicrobial Susceptibility Testing

Antimicrobial susceptibility testing was carried out using the broth microdilution method according to the protocol of CLSI guidelines (CLSI, 2020). Minimum inhibitory concentrations (MICs) were interpreted according to the guideline document established by Clinical and Laboratory Standards Institute (CLSI, 2020). For tigecycline and polymyxin E, the MIC results were categorized in accordance with the breakpoints defined by the European Committee on Antimicrobial Susceptibility Testing criteria^2^. *E. coli* ATCC 25922 was used as a quality control strain.

### S1-PFGE and Southern Blot Hybridization

The plasmid location of the *bla*_*KPC–*__2_ gene was determined by Southern blot experiments according to the previous study (Wang et al., 2020). Briefly, whole chromosomal DNA was digested with S1-nuclease (TaKaRa, Japan). The digested fragments were electrophoresed on a CHEF-mapper XA pulsed-field gel electrophoresis (PFGE) system (Bio-Rad, United States) for 18 h at 14°C. The DNA fragments were transferred to a positively charged nylon membrane (Millipore, United States) and then hybridized with a digoxigenin-labeled *bla*_*KPC–*__2_-specific probe. The fragments was detected by an NBT/BCIP color detection kit (Roche, Germany). The *Salmonella enterica* serotype *Braenderup* H9812 was used as the size marker.

### Conjugation Experiments

A filter-mating experiment was performed with *E. coli* J53 as the recipient strain and *bla*_*KPC*__–__2__–_positive isolates as the donor strains. Transconjugants were selected on Mueller-Hinton agar plates supplemented with 300 mg/L sodium azide and 100 mg/L ampicillin. The transconjugants were confirmed by PCR sequencing and antimicrobial susceptibility testing.

### Whole Genome Sequencing and Plasmid Analysis

Total genomic DNA extraction and analysis were carried out according to previously described methods (Wang et al., 2020). Briefly, the QIAamp DNA MiniKit (Qiagen, Valencia, CA, United States) was used to extract the genomic DNA of two strains for genome sequencing. A NextEra XT DNA library preparation kit (Illumina, Inc., Cambridge, United Kingdom) was used to prepare the DNA library. Genomic DNA was sequenced on an Illumina HiSeq^TM^ 4000 instrument with a 150-bp paired-end approach at a depth of approximately 200×. The CLC Genomics Workbench 10.0 was used to assemble the raw reads of the strains into draft genomes using. In addition, a Pacific Biosciences RSII DNA sequencing system (PacBio, Menlo Park, CA, United States) was used to obtain the complete genomes of strains EC2341 and EC2547. The resulting sequences were *de novo* assembled using the Hierarchical Genome Assembly Process (HGAP_Assembly.2) with the default settings of the SMRT Analysis v2.3.0 software package.

The Rapid Annotation using Subsystems Technology (RAST) annotation website server^3^ was used to annotate the genomes. A schematic map of the linear comparison of the two *bla*_*KPC–*__2_-positive plasmids and their related plasmids was generated with EasyFig 2.2.2 (Sullivan et al., 2011). Multi-locus sequence typing (MLST) of the strain and incompatibility typing of the *bla*_*KPC*__–__2__–_positive plasmid were performed with the assistance of the PlasmidFinder-1.3 server and the MLST 2.0 server, which are available at the Center for Genomic Epidemiology^4^.

In addition, plasmid stability was determined according to a previous study (Li et al., 2018).

### Nucleotide Sequence Accession Number

The complete sequences of the plasmids pEC2341-KPC (accession number CP072979) and pEC2547-KPC (accession number CP072981) were deposited in DDBJ/EMBL/GenBank.

## Results and Discussion

### Isolate Characteristics

In the present study, two KPC-2-producing isolates were collected from a teaching hospital in Zhejiang, China. *E. coli* strains EC2341 and EC2547 were isolated from urine and sputum, respectively. The antimicrobial susceptibility testing results showed that the *bla*_*KPC–*__2_-positive isolates were resistant to carbapenems, cephalosporins, amoxicillin/clavulanate, ciprofloxacin, and amikacin but were susceptible to colistin, tigecycline and ceftazidime-avibactam (Table 1).

**TABLE 1:** Antibiotic susceptibility used in this study (mg/L).

**Strains**	**AMC**	**FEP**	**CAZ**	**ETP**	**IPM**	**MEM**	**CZA**	**AMK**	**CIP**	**TGC**	**CST**
EC2341	128	>128	>128	64	8	16	0.25	4	>128	<0.0625	0.125
EC2341-J53	64	>128	32	64	4	8	<0.125	4	1	<0.0625	0.25
EC2547	128	>128	>128	>64	8	32	0.125	8	>128	<0.0625	0.125
*E. coli* ATCC 25922	4	0.125	0.125	0.125	0.125	0.125	<0.125	0.5	0.125	0.125	0.125

*Drug susceptibility was determined with broth microdilution method according to the Clinical Laboratory Standards Institute (CLSI) guidelines. AMC, amoxicillin clavulanate; FEP, cefepime; CAZ, ceftazidime; ETP, ertapenem; IPM, imipenem; MEM, meropenem; CZA, ceftazidime-avibactam; AMK, amikacin; CIP, ciprofloxacin; TGC, tigecycline; CST, colistin.*

The MLST results showed that *E. coli* strains EC2547 and EC2341 belonged to ST131 and ST410, respectively. The ST131 clone-type *E. coli* strain emerged in the mid-2000s and has spread worldwide (Can et al., 2015). Similar to clone lineage of ST131, the *E. coli* ST410 strain has been confirmed as another successful clone in *E. coli* (Schaufler et al., 2016). Furthermore, these two clone-type *E. coli* strains have gained a further selective advantage due to acquisition of carbapenem resistance (Du et al., 2020; Lee and Choi, 2020). In addition, other resistance genes, such as *bla*_*CTX–M–*__3_, *bla*_*CTX–M–*__27_, *fosA3*, and *qnrS1*, were also detected in the *E. coli* strains by analysis of the genome sequences. Multiple resistance genes were identified in the ST410 and ST131 strains, indicating that these two clone-type strains might be more capable of acquiring resistance genes.

Notably, these two international high-risk clones have caused a wide variety of clinical infections (Roer et al., 2018; Wang et al., 2020) and are associated with treatment failure because of their high virulence potential (Can et al., 2015). In the present study, multiple potential virulence factors were identified by VirulenceFinder analysis of *E. coli* EC2341 and EC2547 strains, such as *ompA* (outer membrane protein A), *fdeC* (adhesin), and *fepC* (iron-enterobactin transporter). *bla*_*KPC–*__2_ was present in the ST131 and ST410 strains, further supporting the results that these two clone types may become a successful lineage of KPC-2-producing *E. coli* strains.

### IncU-Type Plasmid Carrying the *bla*_*KPC–*__2_ Gene

To ascertain the plasmid location of the *bla*_*KPC–*__2_ gene, S1-PFGE was performed followed by Southern blot experiments. The *bla*_*KPC–*__2_ gene was located on two plasmids of different sizes, ca. 80 Kb and ca. 100 Kb (data not shown). The transferability of the two *bla*_*KPC–*__2_-positive plasmids was further determined by filter mating experiments. The EC2341 isolate tested could successfully transfer its carbapenem-resistance to *E. coli* strain J53 (Table 1), while the EC2547 isolate could not transfer its carbapenem resistance. Additionally, the *bla*_*KPC–*__2_-positive plasmids were both stable in the two isolates by plasmid stability experiments. In the absence of antibiotics, the randomly selected strains all carried the *bla*_*KPC–*__2_-positive plasmid that was identical to the parental isolate after 12 rounds of subculture on MH agar.

Incompatibility plasmid classification showed that the two *bla*_*KPC–*__2_-positive plasmids were both grouped into IncU replicon types. The IncU plasmid incompatibility group was assigned in 1981 (Sirgel et al., 1981) and is a unique group of mobile elements with highly conserved backbone functions and variable antibiotic resistance gene cassettes (Tschäpe et al., 1981; Rhodes et al., 2000). The IncU incompatibility group has been isolated from a number of *Aeromonas* spp. and *E. coli* strains from natural and clinical environments (Tschäpe et al., 1981; Sandaa and Enger, 1994; Adams et al., 1998; Rhodes et al., 2000). Various resistance genes have also been described for IncU plasmids, such as *qnrS2*, *aac(6′)-Ib-cr*, *aadA1* and *aadA2, sulI* and *sulII, dfrA16 dfrIIc (dfrB3)* and *catAII* (Sørum et al., 2003). However, carbapenem-resistant IncU plasmids have not been found previously. In this study, the *bla*_*KPC–*__2_ gene was confirmed to be carried on the IncU plasmids. To the best of our knowledge, this is the first report of *E. coli* strains carrying the *bla*_*KPC–*__2_ gene on IncU plasmids. Our study further demonstrated that plasmids harboring the *bla*_*KPC–*__2_ gene were diverse.

### Sequence Analysis of *bla*_*KPC–*__2_ IncU Plasmids

**FIGURE 1:**
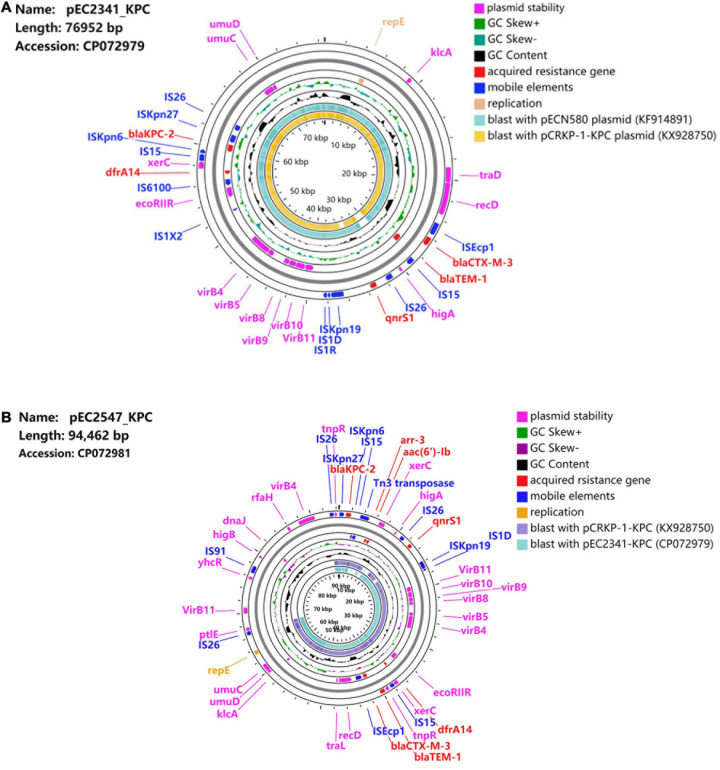
**(A)** Schematic map of plasmid pEC2341_KPC. Sequence alignment between pECN580 (accession number KF914891) and pCRKP-1-KPC (accession number KX928750) is the outer circle in orange and teal. **(B)** Schematic map of plasmid pEC2547_KPC. Sequence alignment between pEC2341-KPC (accession number CP072979) and pCRKP-1-KPC (accession number KX928750) is the outer circle in teal and blue.

**FIGURE 2:**
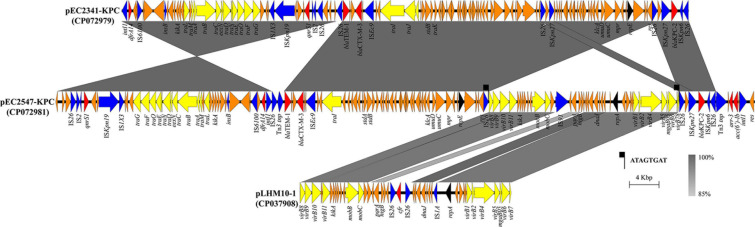
Linear characterization of the plasmids pEC2341-KPC and pEC2547-KPC with closely related plasmid. The gray regions between plasmids indicate nucleotide identity (85–100%) by BLASTn. Arrows indicate predicted ORFs. Primary structural characteristics of plasmids pEC2341-KPC (CP072979), pEC2547-KPC (CP072981), and pLHM10-1 (CP037908). Colored arrows represent open reading frames, with red, yellow, blue, and orange representing antibiotic resistance gene, plasmid transfer related genes, mobile elements and common function genes, respectively.

Two entire sequences were obtained to further characterize the IncU plasmids carrying *bla*_*KPC–*__2_. Sequence analysis showed that plasmid pEC2341_KPC was 76,952 bp in size, had 51.9% G + C content, and harbored 133 predicted ORFs (Figure 1A). The core region of pEC2341_KPC includes a replication module (*repE*), one transfer (*tra*) system, and a stability operon *(stbAB* and *umuCD*). Four antimicrobial resistance genes, *qnrS1*, *bla*_*CTX–M–*__13_, *bla*_*TEM–*__1_, and *drfA14*, were detected in this plasmid except for the *bla*_*KPC–*__2_ gene. In addition, a class 1 integron-like element was also detected in this plasmid. The element is a *dfrA14* gene with its 3′-conserved sequence truncated by the insertion of an *IS*6100 element. Sequence alignments revealed that the plasmid sequences were almost identical to those previously reported plasmids pECN-580 (KF914891) of *E. coli* ECN580 (97% coverage, 99.97% identity) in China (Chen et al., 2014c) and pCRKP-1-KPC (KX928750) of *K. pneumoniae* CRKP-1-KPC (96% coverage, 99.90% identity) in China (unpublished data) (Figure 2).

Plasmid pEC2547 contained *bla*_*KPC–*__2_ and was 94,462 bp in size, with an average G + C content of 49.3% (Figure 1B). Compared with plasmid pEC2341_KPC, two other antimicrobial resistance genes, *aar-3* and *acc(6′)Ib*, were identified in this plasmid. Two class 1 integron-like elements were identified in pEC2547_KPC. The first element is same as that in pEC2341_KPC. The second element is an *IntI1*-*aac(6′)-Ib-cr*-*aar-3-Tn3* gene cassette located downstream of the *bla*_*KPC–*__2_ gene.

Notably, sequencing analysis further indicated that pEC2547 might evolve from plasmid pEC2341_KPC of *E. coli* EC2341 (67% coverage, 100% identity) (Figure 2). Compared with plasmid pEC2341_KPC, an approximately 24-kb sequence flanked by two *IS*26 elements was carried on plasmid pEC2547, which resulted in disruption of the transfer systems of this plasmid. Consistent with our conjugate transfer results, the EC2547 isolate could not transfer its carbapenem resistance to *E. coli* strain J53. The 24-kb sequence was further aligned to an unnamed plasmid of *E. coli* strain LHM10-1 (GenBank accession number CP037908) with 89% coverage and 96.52% identity. This 24-kb composite transposon-like element flanking with two IS26 elements undergoes replicative transposition by the 8-bp target site duplication (TSD) (ATAGTGAT). *IS*26 elements have been demonstrated to undergo frequent intramolecular transposition and facilitate recombination between the plasmid or the chromosome (He et al., 2015). These findings suggest that the plasmid pEC2547 was composed of the pEC2341_KPC plasmid and an unnamed plasmid of *E. coli* strain LHM10-1, which was a composite transposon formed by *IS*26 (Figure 2).

In addition, the *bla*_*KPC–*__2_ gene carried on the two plasmids was preceded by *IS*26, *IS*Kpn27, and *IS*Kpn6, and followed by *IS*26. In China, *bla*_*KPC–*__2_ genetic environments can be classified into three main types: Tn4401 with the *IS*Kpn7-*bla*_*KPC–*__2_-*IS*Kpn6 core structure, Tn1722-based unit transposons with the *IS*Kpn27-*bla*_*KPC–*__2_-*IS*Kpn6 core structure and *IS*26-based composite transposons with the *IS*Kpn27-*bla*_*KPC–*__2_-*IS*Kpn6 core structure (Wang et al., 2015). In this study, *bla*_*KPC–*__2_ genes were both located in an approximately 5-kb composite transposon-like element with the *IS*Kpn27 insertion sequence upstream and the *IS*Kpn6 insertion sequence downstream of the element and flanked by two IS26 elements bracketed by *IS*26, which belonged to the *IS*26-based composite transposon. *IS*26-based composite transposons are mainly carried by IncN-type plasmids. Our plasmids belonged to the IncU type, which led to speculation that the *IS*26 elements may promote recombination between the plasmids and explain the movement of the new IncU regions.

## Conclusion

Overall, we describe here the complete sequences of two novel *bla*_*KPC–*__2_-positive IncU plasmids from *E. coli* isolates. The two *bla*_*KPC–*__2_-harboring plasmids have a close evolutionary relationship, which highlighted the diversity of these highly promiscuous plasmids. The spread of *bla*_*KPC–*__2_ harboring multidrug-resistant plasmids, e.g., pEC2341-KPC and pEC2547-KPC, into the international high-risk clones *E. coli* ST131 and ST410, presents tremendous challenges for clinicians. It is important for the IncU-type plasmid to further disseminate *bla*_*KPC–*__2_ in *Enterobacteriaceae* in order for it to be maintained. Therefore, effective measures should be taken immediately to prevent the spread of these *bla*_*KPC–*__2__–_positive plasmids.

## Data Availability Statement

The complete sequences of the plasmids pEC2341-KPC (accession number CP072979) and pEC2547-KPC (accession number CP072981) were deposited in DDBJ/EMBL/GenBank).

## Ethics Statement

The Ethics Committee of the Zhejiang Provincial People’s Hospital exempted this study from review because the present study focused on bacteria.

## Author Contributions

XL and HP conceived and designed the experiments. WW, LL, and WF performed the experiments. CC and DJ analyzed the data. WW and XL wrote the manuscript. All authors read and approved the final manuscript.

## Conflict of Interest

LL was employed by company Adicon Clinical Laboratories. The remaining authors declare that the research was conducted in the absence of any commercial or financial relationships that could be construed as a potential conflict of interest.

## Footnotes

1
http://www.ncbi.nlm.nih.gov/pathogens/submit_beta_lactamase/

2
http://www.eucast.org/clinical_breakpoints

3
https://rast.nmpdr.org/

4
http://www.genomicepidemiology.org/
