# Corrigendum: Complete Genome Sequences of Two Novel KPC-2-Producing IncU Multidrug-Resistant Plasmids From International High-Risk Clones of *Escherichia coli* in China

**DOI:** 10.3389/fmicb.2021.756781

**Published:** 2021-08-26

**Authors:** Wenhao Wu, Lingling Lu, Wenjia Fan, Chun Chen, Dazhi Jin, Hongying Pan, Xi Li

**Affiliations:** ^1^Department of Infectious Diseases, Zhejiang Provincial People's Hospital, People's Hospital of Hangzhou Medical College, Hangzhou, China; ^2^Medical College, Qingdao University, Qingdao, China; ^3^Adicon Clinical Laboratories, Hangzhou, China; ^4^Department of Pneumology, Zhejiang Provincial People's Hospital, People's Hospital of Hangzhou Medical College, Hangzhou, China; ^5^Centre of Laboratory Medicine, Zhejiang Provincial People's Hospital, People's Hospital of Hangzhou Medical College, Hangzhou, China

**Keywords:** *E. coli*, KPC-2, IncU plasmid, high-risk clones, whole genome sequencing

In the published article, there was an error in affiliation of the authors: Wenjia Fan and Hongying Pan. Instead of “**Wenhao Wu**^**1,2**^^†^**, Lingling Lu**^**3**^^†^**, Wenjia Fan**^**2**^**, Chun Chen**^**4**^**, Dazhi Jin**^**5**^**, Hongying Pan**^2*^and Xi Li^5*^,” it should be “**Wenhao Wu**^**1,2**^^†^**, Lingling Lu**^**3**^^†^**, Wenjia Fan**^**1**^**, Chun Chen**^**4**^**, Dazhi Jin**^**5**^**, Hongying Pan**^1*^ **and Xi Li**^5*^.”

The authors apologize for this error and state that this does not change the scientific conclusions of the article in any way. The original article has been updated.

## Publisher's Note

All claims expressed in this article are solely those of the authors and do not necessarily represent those of their affiliated organizations, or those of the publisher, the editors and the reviewers. Any product that may be evaluated in this article, or claim that may be made by its manufacturer, is not guaranteed or endorsed by the publisher.

